# Scrutinizing Self-Assembly, Surface Activity and Aggregation Behavior of Mixtures of Imidazolium Based Ionic Liquids and Surfactants: A Comprehensive Review

**DOI:** 10.3389/fchem.2021.667941

**Published:** 2021-05-13

**Authors:** Harsh Kumar, Gagandeep Kaur

**Affiliations:** Department of Chemistry, Dr B R Ambedkar National Institute of Technology, Jalandhar, India

**Keywords:** ionic liquids, surfactants, conductance, tensiometer, interaction parameters, mixed micelle

## Abstract

The desire of improving various processes like enhanced oil recovery (EOR), water treatment technologies, biomass extraction, organic synthesis, carbon capture etc. in which conventional surfactants have been traditionally utilized; prompted various researchers to explore the self-assembly and aggregation behavior of different kinds of surface-active molecules. Ionic liquids (ILs) with long alkyl chain present in their structure constitute the advantageous properties of surfactant and ILs, hence termed as surface-active ionic liquids (SAILs). The addition of ILs and SAILs significantly influence the surface-activity and aggregation behavior of industrially useful conventional surfactants. After a brief review of ILs, SAILs and surfactants, the prime focus is made on analyzing the self-assembly of SAILs and the mixed micellization behavior of conventional surfactants with different ILs.

## Introduction

Exploring the self-assembly, surface-activity as well as aggregation behavior of newly synthesized surface-active agents having improved properties is crucial for their utilization in place of traditionally used surfactants to enhance their quality and performance in various processes. Various kinds of surface-active agents include (i) conventional surfactants of various classes based upon their charged head group and (ii) surface active ionic liquids (SAILs) inheriting the unusual properties of both surfactants and ionic liquids (ILs). The structure, unusual properties and vast applicability of ILs have been discussed first to enlighten the scope of ILs. Also, the knowledge of various classes of SAILs and surfactants based upon their structure is essential before understanding their self-assembly and aggregation behavior. Then, general synthesis steps for imidazolium-based ILs have been included along with the introduction of various techniques and theoretical models, which have been potentially utilized to determine the aggregation behavior of various surface-active agents as well as their mixtures in aqueous media. The surface-activity and aggregation behavior get significantly influenced by the addition of any additive in the system at various concentrations (Alam et al., 2016; Azum et al., 2018; Das et al., 2018). Thus, for tuning and designing micellar systems based upon requirement in particular application, it is crucial to explore the mixed micellization behavior of various ILs/surfactant mixtures. The mixed micelles formed by SAILs/surfactant mixtures are observed to be more thermodynamically stable than individual surfactant micelles, leading to their enhanced utility (Dutta et al., 2018; Garcia et al., 2020). A lot of research based upon the self-assembly as well as mixed micellar behavior of SAIL/ surfactant systems have also been systematically reviewed in this article. As per our knowledge, there is no such article available in the literature comprising this set of information in single manuscript, so that the readers who are unfamiliar with the field could easily follow and understand the scope.

### Ionic Liquids (ILs)

**Figure 1:**
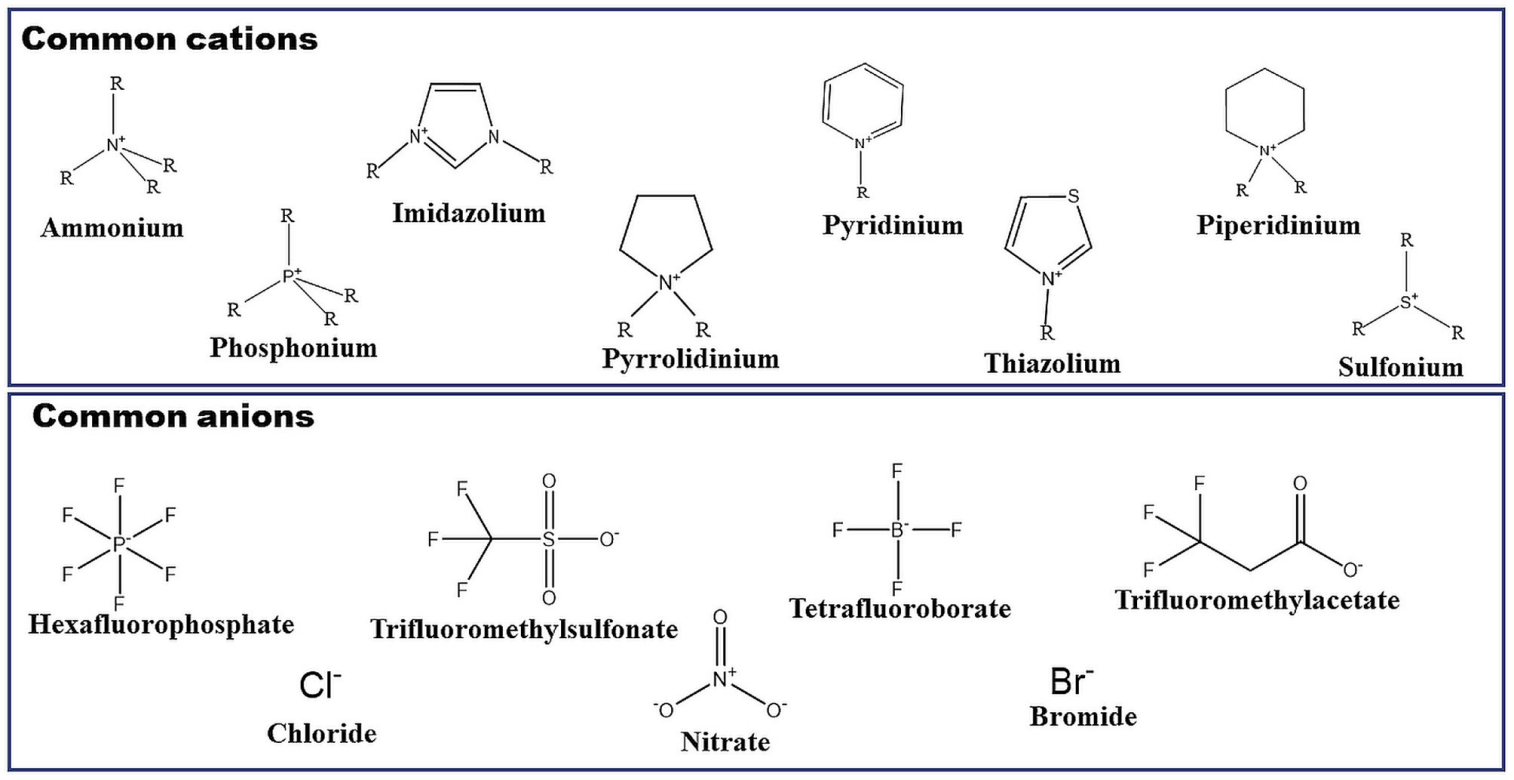
Common cations and anions for the synthesis of ionic liquids (ILs).

ILs can be considered as novel solvents having unique properties. Because of the interesting properties such as their non-volatile nature, good conductivity, good thermal stability, and non-flammability, etc., these liquids are revolutionizing the world of chemistry (Welton, 1999; Brennecke and Maginn, 2001; Rogers and Seddon, 2003; Weyershausen and Lehmann, 2005). They are low melting salts obtained by combining asymmetric and bulky organic cations with various anionic species. There is a lot of such combinations present that could result in the preparation of a vast number of ILs (Earle and Seddon, 2002; Ghandi, 2014; Paul and Moulik, 2015). Figure 1 represents different cationic as well as anionic species which could be combined to synthesize a lot of ILs. Apart from the ionic species shown in Figure 1, there are a lot of other species which can lead to the synthesis of ILs. Based upon the type of cation and anion, ILs can have their unique properties. By analyzing these properties, perfect IL can be chosen for the particular application. ILs can be utilized in a variety of fields such as synthesis of organic and inorganic materials, electrochemistry, catalysis as well as bio-catalysis reactions, materials science, and separation technology (Chotkowski et al., 2020; Gholami et al., 2020; Lee et al., 2020; Nasirpour et al., 2020; Su et al., 2020; Yin et al., 2020). Imidazolium-based ILs have become the focus of much research because of the presence of the imidazole ring in the structure. This ring is present in histidine, which can interact with hemoglobin. Thus, the imidazolium ring is very important from a biological point of view. This is the reason that imidazolium-based ILs having 1-alkyl-3-methylimidazolium as cation is very important and a lot of ILs can be synthesized by simply changing the anion as well as the length of the alkyl chain attached to the imidazolium ring (Bates et al., 2002; Dupont et al., 2002; Wasserscheid and Welton, 2002; Paul et al., 2005; Plechkova and Seddon, 2008; Singh and Kumar, 2008a; Sharma and Mahajan, 2014; Pendleton and Gilmore, 2015; Pal and Punia, 2019c). Imidazolium-based ILs can be of two kinds. One having a short alkyl chain and the other having a long alkyl chain. This alkyl chain can be present in both cationic as well as anionic parts of ILs. For the former case having a short hydrophobic chain present in the structure, there is the formation of polar and non-polar domain nanostructures (Shimizu et al., 2010; Greaves and Drummond, 2013; Jiang et al., 2018) in water. But in the case of a long hydrophobic chain (n>8) present in the structure, the obtained ILs become surface-active and own the property of self-aggregation like conventional surfactants (Paul and Moulik, 2015; Cognigni et al., 2016). This type of ionic liquids is known as surface-active ionic liquids (SAILs), which are discussed in the next section.

### Surface Active Ionic Liquids (SAILs)

**Figure 2:**
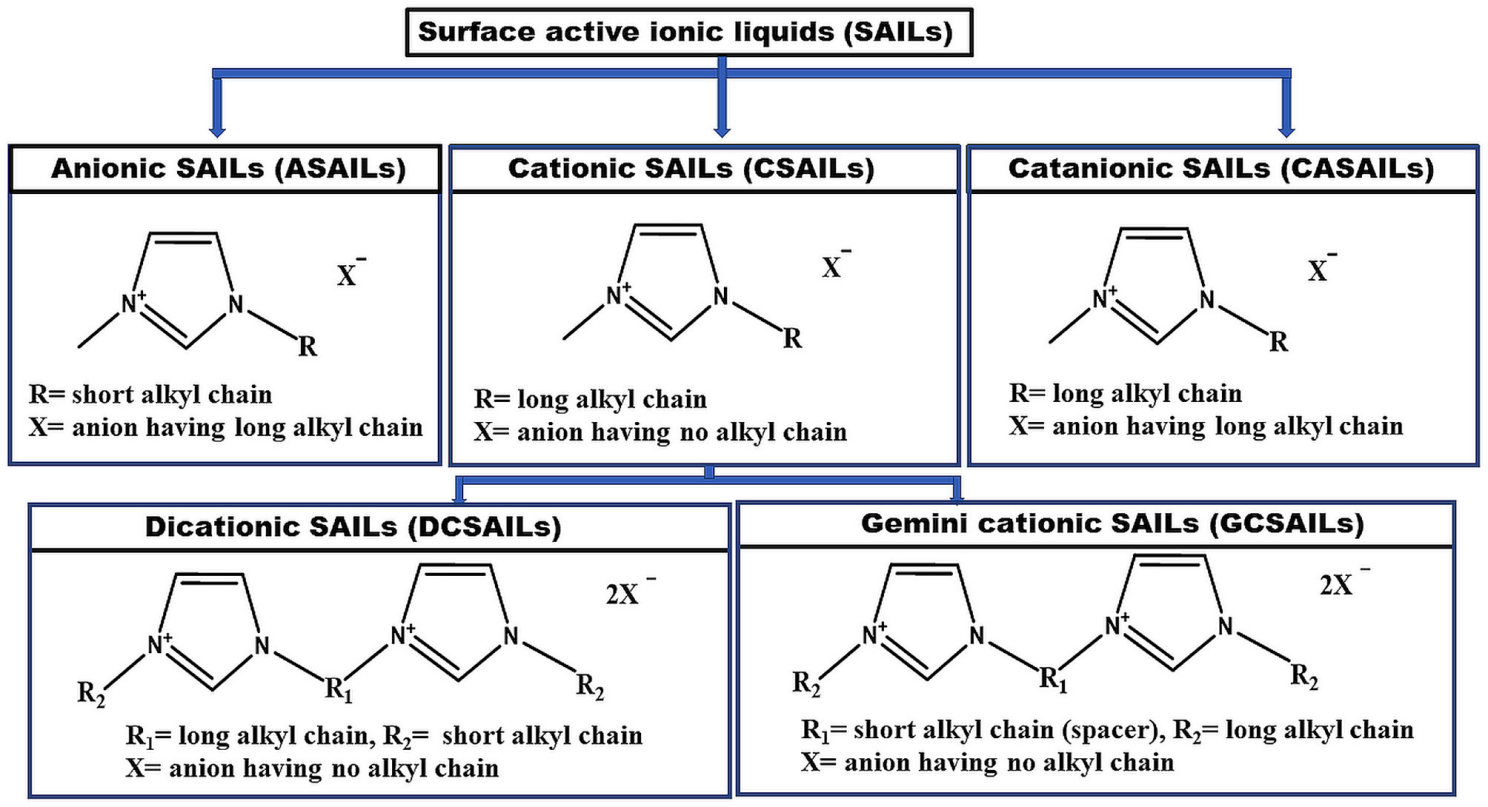
Molecular structures of various types of surface-active ionic liquids (SAILs).

SAILs can undergo micelle formation like conventional surfactants as SAILs are structurally analogous to them. Their cationic or anionic part possesses a charged head group and a long hydrophobic tail in it. This is responsible for the self-aggregation property and surface activity of SAILs (Cobanov et al., 2020; Garcia et al., 2020; Singh et al., 2020; Alves et al., 2021). There is no hardliner difference among SAILs and surfactants. SAILs differ from traditional ionic surfactants in terms of their low melting point (<100°C), surface activity and functionality only. There are many fundamental benefits of SAILs over conventional surfactants such as their liquid nature, low melting point, good solubility in aqueous media, superior activity, lower CMC value as well as tunable nature. Due to the similar amphiphilicity to traditional surfactants, they find their applications as emulsifiers, dispersants, and foaming agents. Task-specific applications of SAILs have led to the design of a large number of surfactant systems based on their self-assembled aggregates such as micelles (wormlike and rod shape), vesicles, liquid crystalline phases, tubules, etc. Vesicles are of utmost significance, not only because they mimic biological membranes but also due to their utility as colloids formulation, material synthesis, nutrient transportation, cell signaling, DNA protection, as drug carriers and targeted drug delivery systems (Lépori et al., 2019; Garcia et al., 2020). Micellar catalysis is another purpose served by aggregates of SAILs (Cognigni et al., 2016, 2017). Their hydrophobic core in water acts as an efficient micellar nano-reactor. The use of SAILs as catalyst exhibits a dramatic kinetic effect on the rate of reaction, percentage yield of product for various organic reactions. Other applications of SAILs can be listed as foaming and antifoaming agents, antimicrobials, demulsification of crude oil, chromatographic and electrophoretic separations, enhanced oil recovery (EOR), solubilization of drugs, extraction of natural products etc. (Shah et al., 2018a, 2020; Vaid et al., 2018a,b; Dani et al., 2019; Isosaari et al., 2019; Kuddushi et al., 2019, 2020; Shakeel et al., 2019; Lee et al., 2020; Nandwani et al., 2020; El Seoud et al., 2021). SAILs can be of different kinds based upon their structure. The main three types of SAILs are cationic surfactant-like ILs (CSAILs), anionic surfactant-like ILs (ASAILs), and catanionic surfactant-like ILs (CASAILs). In the case of CSAILs, the long hydrophobic chain is present in the cationic part of SAIL, giving rise to similar properties like conventional cationic surfactants (Zhao and Zheng, 2011; Gu et al., 2013; Cognigni et al., 2016; Zhao et al., 2017). CSAILs can be subdivided into two types namely dicationic surface-active ILs (DCSAILs) and Gemini cationic surface-active ILs (GCSAILs) (Baltazar et al., 2007; Ao et al., 2008; Khan et al., 2017; Ziembowicz et al., 2017; Zhou et al., 2018). Similarly, if the long hydrophobic chain is present in the anionic part of SAIL exhibiting the properties similar to anionic surfactants, then they will be considered as ASAILs (Jiao et al., 2012, 2013b; Srinivasa Rao et al., 2014). If the long alkyl chain is present in both cationic and anionic parts of SAILs, then it will result in the new category of SAILs termed as CASAILs (Jiao et al., 2013a; Sohrabi et al., 2015; Zheng et al., 2018). Figure 2 indicates molecular structures of various types of SAILs. These SAILs are found to be more advantageous than traditional surfactants due to their higher surface activity as well as thermodynamically and kinetically stable micelle formation than that observed in the case of self-assembly of surfactant molecules.

### Surfactants

**Figure 3:**
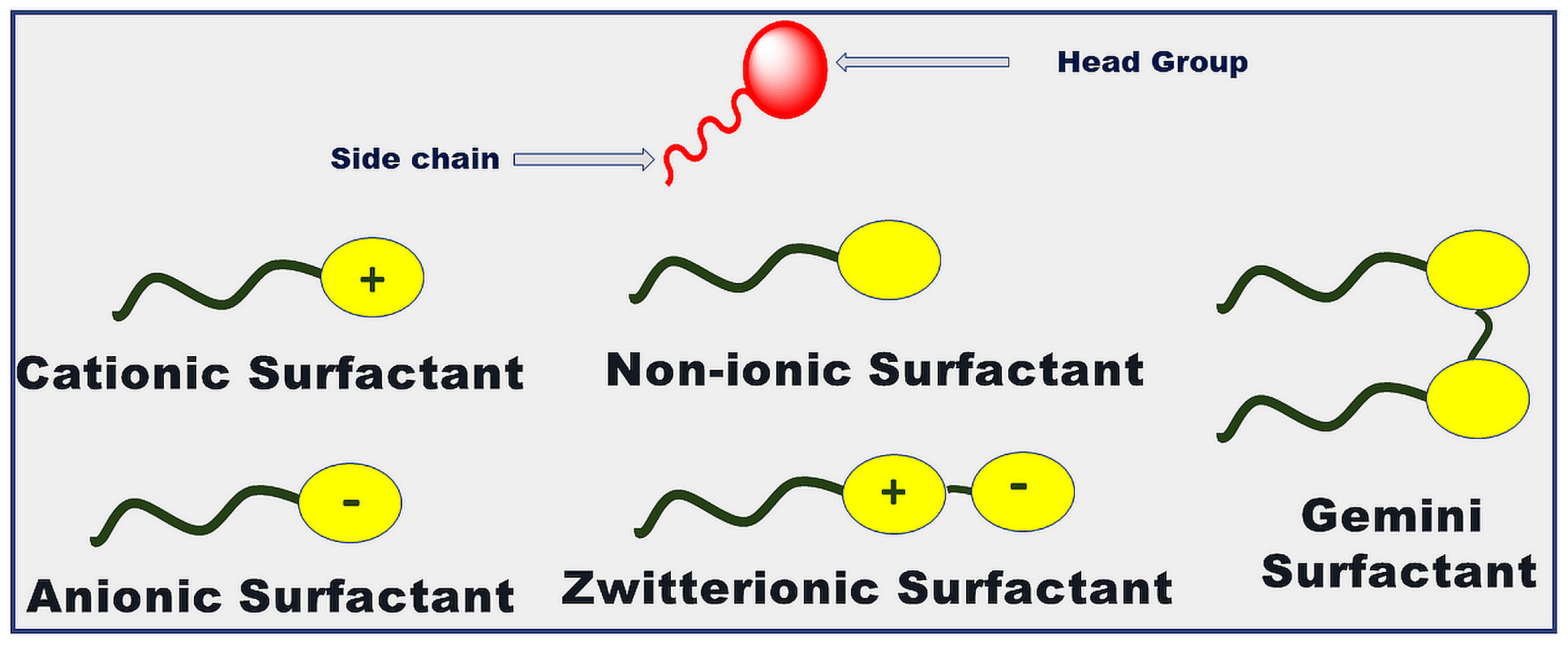
Structure of surfactant with its various types.

Surfactants are surface-active agents having the unique property to adsorb at the interface. This is due to the amphiphilic nature of surfactant monomers. They have a polar head group and non-polar hydrophobic tail present in their structure as shown in Figure 3. The surfactant monomers can aggregate to form colloidal-sized clusters (1–100 nm) in solutions, known as micelles. The formation of micelle occurs over a sharp range of concentrations of surfactant known as critical micelle concentration (CMC) due to the delicate balance between hydrophobic and hydrophilic interactions. The surfactant monomers are dispersed in solution below this concentration (Moroi, 1992; Rosen and Kunjappu, 2012). Due to this property of self-aggregation, they have been used extensively in a variety of areas for decades. They find their applications as detergents for cleaning processes, lubricants for automobiles, in electronic printing, biotechnology, cosmetics, petroleum industries, etc. (Moroi, 1992; Kumar et al., 2018b; Kumar and Rub, 2019; Sagir et al., 2020). The excretion of hazardous heavy metals and dyes from industries or households leading to groundwater contamination can cause a lot of serious as well as long term toxicity effects (Sharma et al., 2014; Naushad and ALOthman, 2015; Naushad et al., 2015a, 2016, 2019a,b; Tatarchuk et al., 2019). But the surfactants have been employed to remove such contamination of ground water through surfactant-assisted cation exchangers leading to enhance the applicability of surfactants (Naushad, 2014; Naushad et al., 2015b; Muthusaravanan et al., 2018; Mironyuk et al., 2019; Ahmed et al., 2020; Balasubramani et al., 2020; Faisal et al., 2020).

**Figure 4:**
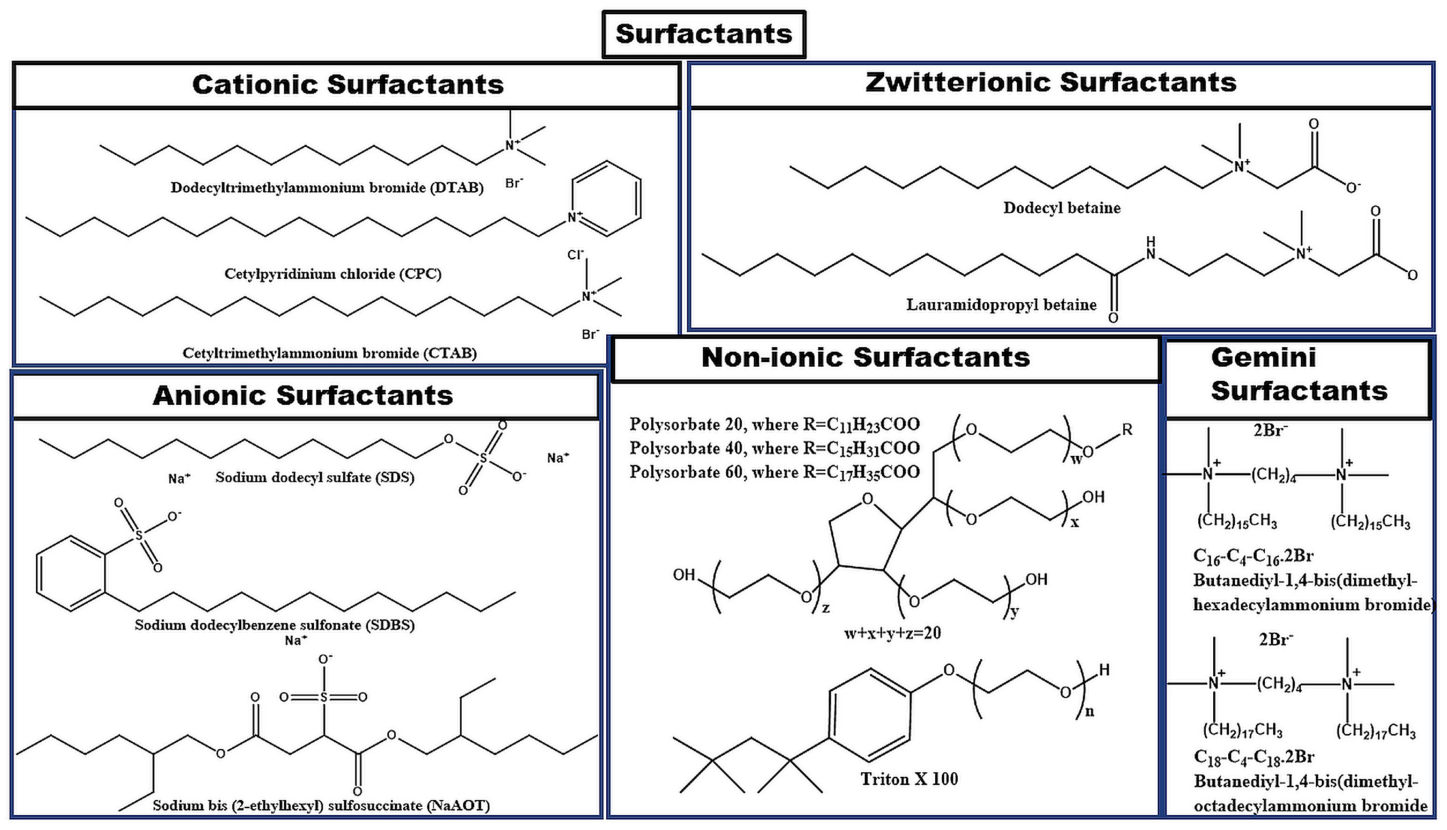
Examples of various types of surfactants.

Surfactants can be classified into various types depending upon the composition of the head group as shown in Figure 3. It can be observed from the figure that if the surface-active part of surfactant monomers owns a positive charge, then the corresponding surfactant is termed as cationic surfactant. If the negative charge is there on the surface-active part, then the surfactant is an anionic surfactant in nature. If the surface-active part of the surfactant holds both the positive as well as the negative charge on it, then it is termed as zwitterionic surfactant. If no charge is present on the surface-active part of the surfactant, then it is termed as a non-ionic surfactant. In the case of Gemini surfactant, two traditional surfactant monomers are bonded together through a spacer group. Gemini surfactants can have short or long hydrophobic tails; cationic, anionic, or non-ionic polar head groups and short or long or flexible or rigid spacer groups present in its structure (Menger and Keiper, 2000; Kumar and Rub, 2019; Liao et al., 2019). Figure 4 incorporates the examples of common conventional surfactants along with their type to provide knowledge on surfactants.

## Experimental

### Synthesis of Imidazolium-Based ILs

**Figure 5:**
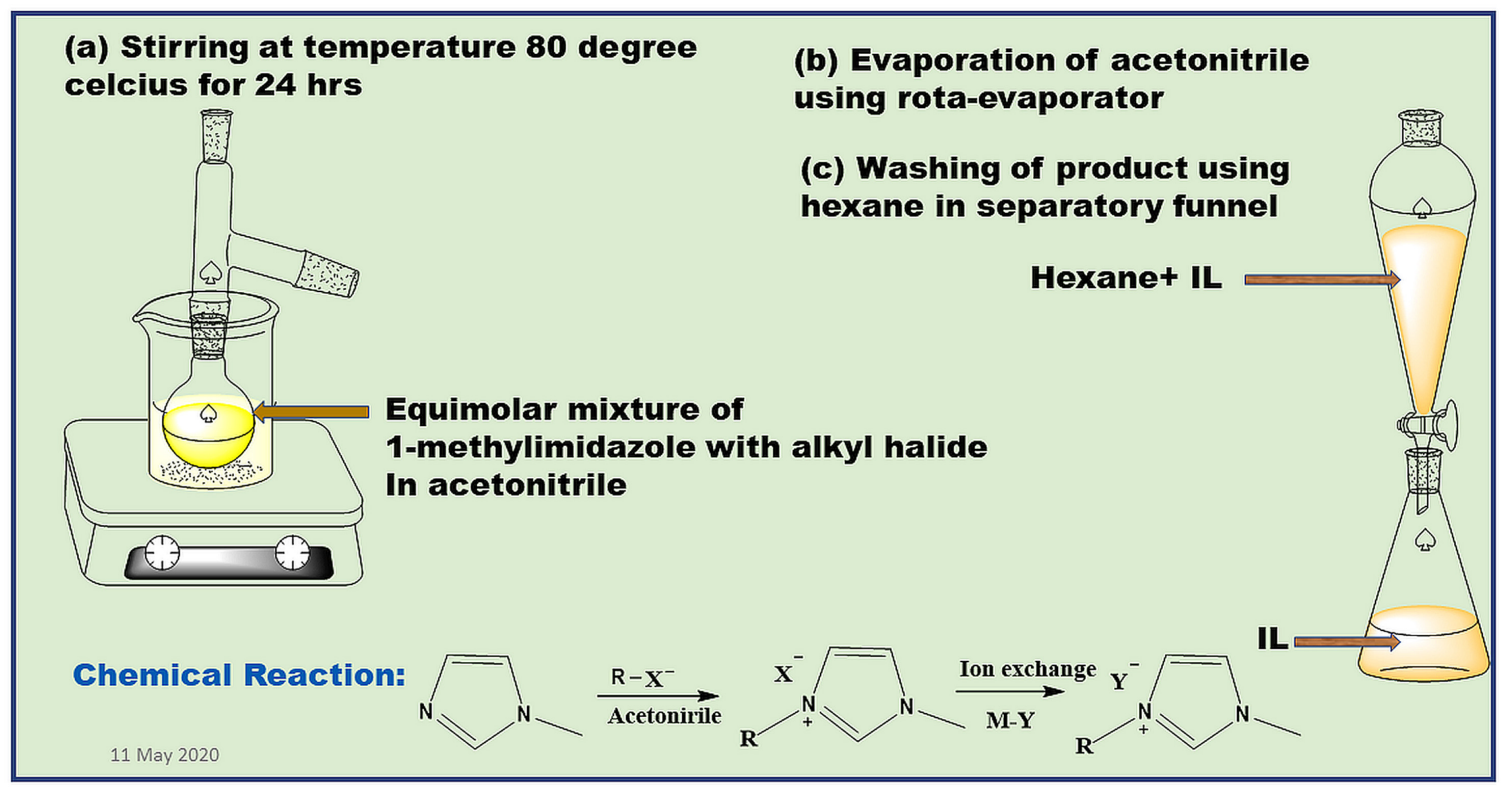
General steps for synthesis of ionic liquid.

The synthesis of imidazolium-based ILs can be done by following two distinct steps utilizing bimolecular nucleophilic substitution (SN2) reaction. In the first step, a quaternization reaction is performed in which alkylation of 1-methylimidazole is done by using alkyl halide, and organic halide salt is formed (Cristina, 2014; Paul and Moulik, 2015). This can be done by adding haloalkane into 1-methylimidazole dropwise in the round-bottomed flask in the ratio of 1:1.2. Then the reaction mixture was dissolved in solvent mainly acetonitrile. The mixture so obtained is then refluxed for 48 h at about 80–84?C temperature with proper stirring of the reaction mixture. Thin layer chromatographic technique is followed to monitor the progress of the reaction. The reaction mixture was then cooled and acetonitrile was evaporated using a rotary evaporator. Then the washing of the resulting mixture is done using hexane to remove any impurities present in it (Paulechka et al., 2007; Sun et al., 2014; Kumar and Kaur, 2020a). The product so obtained is 1-alkyl-3-methylimidazolium halide, which can be considered as an ionic liquid having halide ion as its counter ion as shown in Figure 5. The whole procedure of alkylation of 1-methylimidazole can be done in the absence of solvents by employing microwave or ultrasound irradiation (Khadilkar and Rebeiro, 2002; Aupoix et al., 2010). The next step involves the metathesis of halide ion in the resulting product. For this ion exchange reaction is done. This can be done by employing a microporous ion exchange column or by reacting the 1-alkyl-3-methylimidazolium halide with the compound having the required counterion. In the case of dodecyl sulfate-based counterions, the reaction can be performed by stirring the components i.e., 1-alkyl-3-methylimidazolium halide and sodium dodecyl sulfate in dichloromethane at room temperature for at least 8 h. The precipitates obtained are then removed using filtration and washing of the organic phase was done using water until the water was halide-free. The product so obtained is dried in a vacuum for few days (Jiao et al., 2012, 2013b; Xu et al., 2015). To know whether the desired ionic liquid has been synthesized or not, its characterization can be done using ^1^H-NMR, ^13^C-NMR, and FTIR spectroscopic techniques. If the results obtained using these spectral studies were found to be in good agreement with the desired ionic liquid, only then it is utilized for further micellization studies. The sonochemical synthesis pathway can also be applied to prepare imidazolium-based ILs, which is a green ultrasound-assisted process as described by Ameta et al., 2015.

### Micellization Study

**Figure 6:**
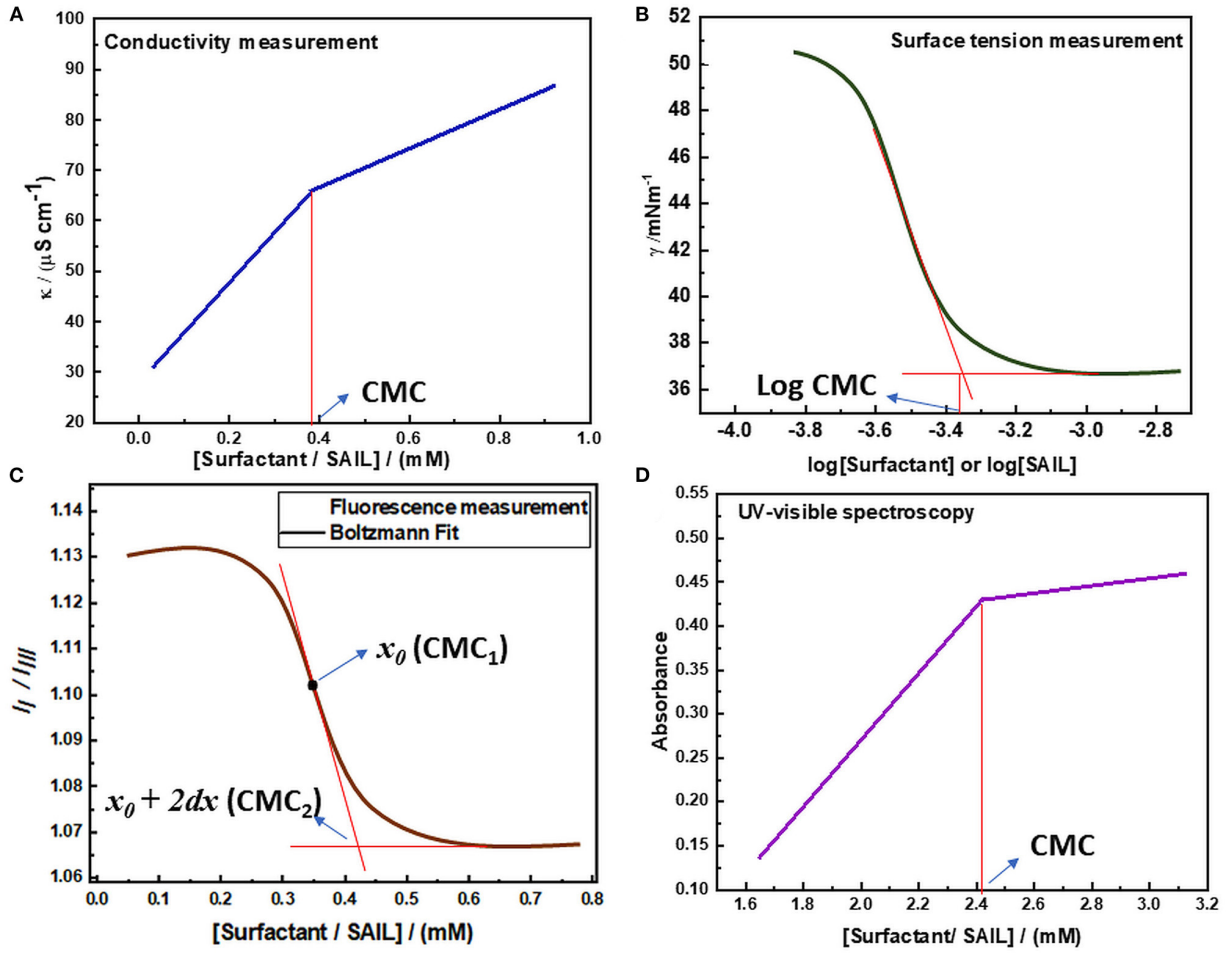
Determination of CMC via **(A)** Conductivity measurement **(B)** Surface tension measurement **(C)** Fluorescence measurement **(D)** UV-visible spectroscopy.

The amphiphilic molecules i.e., surfactants or SAILs can do micelle formation beyond a particular concentration. Several properties such as conductivity, surface tension, refractive index, etc. show a sharp change in the vicinity of this concentration. This fact has been utilized by various researchers to determine the value of CMC. Apart from the value of CMC, various other important parameters such as thermodynamic parameters of micellization, mixed micellar parameters, surface-active parameters, shape, and size of a micelle formed can also be determined to give insight into the process of micelle formation. Various common techniques employed for the investigation of self-assembly and aggregation of surface-active molecules have been discussed in detail. Various types of plots obtained by different techniques have been shown in Figure 6.

#### Conductivity Measurements

The digital conductivity meter was employed to know the specific conductivities of the solution. The measurement is done by accurately maintaining the temperature of the solution by using a refrigerated circulated water thermostat as the values of specific conductivity are dependent on temperature and vary with the variation in temperature of the system. The specific conductivity measurement was carried out for various concentrations of solution in triplicate and the mean of them was considered as the final result. The final values of specific conductivities obtained were plotted against the concentration of surfactant in solution to determine the value of CMC of that surfactant as shown in Figure 6A. The obtained graph indicates that the values of specific conductivity increase with the increase in the concentration of amphiphilic molecules. But after the micelle formation, the rate of increase in conductivity decreases because the formed micelles have lower mobility than the distinct monomeric surfactant molecules that were present before. Thus, a breakpoint arises in the line and the concentration of surfactant corresponding to this breakpoint gives the value of CMC (Bachofer, 1996; Jungnickel et al., 2008; Ali et al., 2014, 2016; Chauhan and Sharma, 2014). This method can also be utilized to determine the value of the degree of counterion dissociation as well as various thermodynamic parameters of micellization. The degree of counterion dissociation (g) can be calculated by using equation (1).

(1) $$\begin{array}{l} g=\frac{S_{2}}{S_{1}} \end{array}$$

where *S*_1_ is the slope of the pre-micellar region and *S*_2_ is the slope of the post-micellar region in the plot of specific conductivity vs. concentration of the surface-active agent.

The temperature dependence of CMC has been utilized to obtain various thermodynamic parameters of micellization by using the following equations.

(2) $$\begin{array}{l} \Delta G_{m}^{0}=\left(2-g\right)RT\;\left(\ln X_{CMC}\right) \end{array}$$

(3) $$\begin{array}{l} \Delta H_{m}^{0}=-RT^{2}\;\left(2-g\right)\left[\frac{d\left(\ln\;X_{CMC}\right)}{dT}\right] \end{array}$$

(4) $$\begin{array}{l} \Delta S_{m}^{0}=\left(\Delta H_{m}^{0}\;-\;\Delta G_{m}^{0}\right)\;/\;T \end{array}$$

where $\Delta G_{m}^{0}$ is the standard free energy of micellization, ${\Delta H}_{m}^{0}$ denotes standard enthalpy of micellization, $\Delta S_{m}^{0}\;$represents standard entropy of micellization, *R* is gas constant, *T* is temperature, *X*_*CMC*_ denotes the value of *cmc* in the mole fraction unit and *g* signifies the degree of counterion dissociation (Pal and Chaudhary, 2013a,b; Chaudhary and Pal, 2014; Pal and Yadav, 2017a; Azum et al., 2019). The values of these parameters give insight into the driving force behind the process of micellization and tell whether the nature of micellization is enthalpy-driven or entropy-driven.

#### Surface Tension Measurement

The CMC value and surface-active parameters of micellization can be more accurately determined by employing surface tension measurement. This is usually done by employing a well-equipped tensiometer having specially designed software for CMC determination. The more accurate determination of CMC via surface tension measurement is due to the reason that the employed surface tensiometer utilizes an automatic system for the high precision, accurate and reliable measurements of surface tension, which can further lead to determine CMC by plotting surface tension against the logarithm of the concentration of surfactant. Surface tension measurement of solutions at various concentrations can be done either by Wilhelmy plate or Du Noüy ring method at a constant temperature. At least three readings of surface tension are usually taken for specific concentration and the mean of which is considered as the final value. The values of surface tension obtained are then plotted against the logarithm of the concentration of surface-active molecules as shown in Figure 6B. It can be observed from the graph that below the CMC, the surface tension of the solution decreases with increasing the concentration of surface-active molecules. But after the addition of sufficient surfactant monomers, the interface becomes saturated and further addition of surfactant monomers leads to micelle formation. The surface tension of the solution becomes nearly constant above CMC because the concentration of surfactant at the interface does not change due to the saturation of the interface. Thus, the value of CMC can be obtained at the point of intersection obtained by extrapolating the regression lines obtained in the plot of surface tension vs. logarithm of surfactant concentration. Also, various surface-active parameters can be determined by employing the CMC value obtained (Mahajan and Sharma, 2011; Pal and Yadav, 2016; Zhao et al., 2017; Panda et al., 2018; Liao et al., 2019).

The Gibbs adsorption isotherm equation given below can be utilized to determine the maximum surface excess concentration (Γ_max_) and minimum area of surfactant monomer at air/ water interface (*A*_min_) (Patel et al., 2015; Pal and Yadav, 2017b; Shah et al., 2018b; Garcia et al., 2020).

(5) $$\begin{array}{l} \Gamma_{\max}=-\frac{1}{RT}\left(\frac{\partial \gamma }{\partial \left(\ln C\right)}\right) \end{array}$$

(6) $$\begin{array}{l} A_{\min}=\frac{1}{N_{A}\Gamma_{\max}} \end{array}$$

where ∂γ /∂(ln*C*) denotes the maximum slope, *R* is the universal gas constant, *T* is the temperature, γ signifies the surface tension of the solution, *C* denotes to the total concentration of the solution, *N*_*A*_ symbolize the Avogadro's number. The values of these parameters tell us about the packing of surface-active molecules at the interface. The maximum surface excess concentration (Γ_max_) is area related concentration of surfactant at the interface and the minimum area of surfactant monomer at air/ water interface (*A*_min_) can be considered as an effective area of the head group. The larger magnitude of Γ_max_ and smaller value of *A*_min_ indicates more packing of monomers at the interface, whereas the smaller magnitude of Γ_max_ and larger magnitude of *A*_min_ signifies less packing of monomers at the interface. The surface pressure at *cmc* (∏_cmc_) is another important parameter which is the measure of the efficiency of surfactant to lower the surface tension of water. This can be calculated by employing equation (7).

(7) $$\begin{array}{l} {\;}_{cmc}=\gamma_{0}\;-\;\gamma_{cmc} \end{array}$$

where γ_0_ indicates surface tension of solvent in pure state and γ_*cmc*_ symbolize the surface tension of solution at*cmc*.

Adsorption efficiency (*pC*_20_) signifies the tendency of surfactant to adsorb at air/ water interface and can be calculated by using the following equation.

(8) $$\begin{array}{l} {pC}_{20}=-\log C_{20} \end{array}$$

where C_20_ represents the concentration of surfactant molecules required to reduce the surface tension of pure solvent by 20 mN m^−1^.

The standard Gibbs free energy of adsorption ($\Delta G_{ad}^{0}$) can be evaluated by using equation (9), which indicates whether the adsorption of surfactant at air/ water interface is spontaneous or not. The more negative values of $\Delta G_{ad}^{0}$ as compared to $\Delta G_{m}^{0}$ are usually obtained, which indicates that the adsorption of surfactant monomers is the primary process as compared to the process of micellization (Patel et al., 2015; Pal and Yadav, 2017b; Shah et al., 2018b; Garcia et al., 2020).

(9) $$\begin{array}{l} \Delta G_{ad}^{0}=\Delta G_{m}^{0}\;-\;\frac{\prod_{cmc}}{\Gamma_{\max}} \end{array}$$

where $\Delta G_{m}^{0}$ denotes the standard Gibbs free energy of micellization, π_cmc_ is surface pressure at CMC, Γ_max_ is the maximum surface excess concentration.

Another important parameter proposed by Sugihara et al. is the free energy of a surface at equilibrium ($G_{\min}^{s}$), which is utilized to evaluate synergism in the mixed adsorption film at equilibrium (Rosen and Kunjappu, 2012; Chabba et al., 2015; Patel et al., 2015; Pal and Yadav, 2017b; Shah et al., 2018b; Garcia et al., 2020).

(10) $$\begin{array}{l} G_{\min}^{s}=A_{\min}\gamma_{cmc}N_{A} \end{array}$$

where A_min_ is the minimum area per molecule, γ_cmc_ is the surface tension at CMC, N_A_ refers to Avogadro's number.

The packing parameter (*p*) which gives an idea about the shape of the micelle formed can be calculated by using equation (11).

(11) $$\begin{array}{l} p={V_{0}/l_{c}A}_{\min} \end{array}$$

where *V*_0_ signifies the volume occupied by the hydrophobic group in the core of the micelle, *l*_*c*_ denotes the length of the hydrophobic core. The value of *V*_0_ and *l*_*c*_ can be calculated by employing Tanford's formulas given below (Tanford, 1980; Mata et al., 2004; Zhao et al., 2017; Pal and Saini, 2019).

(12) $$\begin{array}{l} V_{0}=\left[27.4+26.9\left(n_{c}-1\right)\right]2\left({À}^{3}\right) \end{array}$$

(13) $$\begin{array}{l} l_{c}=\left[1.54+1.26\left(n_{c}-1\right)\right]\left(À\right) \end{array}$$

where *n*_*c*_ represents the total number of carbons in the hydrocarbon chain. In equations (12) and (13), one carbon atom is substracted from the *n*_*c*_ because the first carbon after the head group has high solvation and is therefore not considered as part of the hydrophobic alkyl chain. If the magnitude of the packing parameter is <0.33, then the shape of the micelle is spherical. If *p* lies between 0.33 and 0.5, the cylindrical micelle is formed. If *p* lies between 0.5 to 1, then bilayer vesicle formation is there in the system. If *p* becomes nearly equal to one, then planar lamellar phases are formed. If *p* comes out to be greater than one, then reverse micelle formation is there in the solution.

#### Fluorescence Measurement

Fluorescence spectroscopy is a type of photoluminescence in which a beam of light excites the electrons in molecules. The electrons in the singlet ground state are promoted to a singlet excited state by the adsorption of photons. The excited electrons, then return to the ground state and emit a photon of lower energy and a longer wavelength than absorbed photons (Kühnemuth and Seidel, 2001; Pal and Pillania, 2016a). Here pyrene is used as a fluorescent probe, which has been added in surfactant solutions in very low concentration to determine the value of CMC. Pyrene *I*_*I*_ */I*_*III*_ method is utilized to determine the CMC of any surfactant, as the fluorescence spectra of pyrene show five vibronic peaks. The intensity of the first and third vibronic peaks is utilized to determine the CMC value, as these peaks are sensitive to the polarity of the medium. The magnitude of *I*_*I*_ */I*_*III*_ changes with the change in polarity of the medium. Its value is more in the polar medium but gets decreased with the decrease in polarity. The abrupt decrease in this ratio is due to the reason that pyrene *I*_*I*_*/I*_*III*_ corresponds to the polar environment before micelle formation. But the hydrophobicity of solution increases with the increase in the concentration of surfactant in solution and the value of pyrene *I*_*I*_ */I*_*III*_ decreases. As the surfactant concentration reaches critical micelle concentration, pyrene goes to the hydrophobic micellar phase and binds to surfactant aggregates, leading to a nearly constant value of pyrene *I*_*I*_ */I*_*III*_ (Kalyanasundaram and Thomas, 1977; Aguiar et al., 2003)_._ Thus, in the plot of pyrene *I*_*I*_ */I*_*III*_ vs. concentration of surfactant, a Boltzmann type sigmoidal curve is obtained as represented in Figure 6C.

(14) $$\begin{array}{l} y=\frac{A_{1}-A_{2}}{1+e^{\left(x-x_{0}\right)/dx}}\;+\;A_{2} \end{array}$$

where *A*_1_ and *A*_2_ are the initial and final asymptotes of the sigmoid, respectively, *x* denotes the concentration of surfactant, *x*_0_ symbolizes the center of the sigmoid, and dx is the interval of the independent variable *x*. These parameters can be ascertained by fitting the data in the Boltzmann curve in Origin software. The two CMC values of a particular surfactant can be obtained by utilizing this method, one at *x*_0_ and the other at *(x*_0_ + *2dx)*. The *x*_0_ is considered as CMC value only if (*x*_0_*/dx)* < 10, whereas *(x*_0_ + *2dx)* is considered as CMC value only if *(x*_0_*/dx)* >10 (Kalyanasundaram and Thomas, 1977; Aguiar et al., 2003; Basu Ray et al., 2006; Kumar and Kaur, 2020b).

This method can be further utilized to determine the aggregation number (*N*_*agg*_) of surfactant micelles. The magnitude of *N*_*agg*_ can be by fluorescence quenching of pyrene. The cetylpyridinium chloride (CPC) is commonly used as a quencher.

(15) $$\begin{array}{l} \ln\left(\frac{I_{0}}{I_{Q}}\right)==\left[Q\right]\left[\frac{N_{agg}}{S-cmc}\right] \end{array}$$

where *I*_0_ and *I*_*Q*_ denotes fluorescence intensities of pyrene in the absence and presence of quencher, respectively. [*Q*] denotes the concentration of quencher, *S* denotes surfactant concentration. The plot of ln(*I*_0_/*I*_*Q*_) *vs*. [*Q*] is having a straight line, whose slope can be utilized to determine the value of *N*_*agg*_ of surface-active molecules (Wang et al., 2007; Pal and Pillania, 2015b, 2017; Piñeiro et al., 2015; Singh and Kang, 2015; Chadha et al., 2016; Banjare et al., 2017; Pal and Punia, 2019b).

#### UV-Visible Spectroscopy

Another important technique that could be utilized to determine the critical micelle concentration value of surface-active molecules is UV-visible spectroscopy. This method is based upon the fact that there is the change in absorption behavior of solution upon aggregation of surface-active molecules. The determination of CMC is done by using an organic dye as a probe, which is having characteristic UV-visible spectra. The change observed in the absorption behavior of that additive may lead to the estimation of critical micelle concentration value. But the use of such kind of additives in the solution of surface-active agents may alter their aggregation process, hence lead to estimation of apparent CMC value. These organic probes can also affect the stability of formed micelles. Thus, this method of estimation of critical micelle concentration is less reliable (Beyaz et al., 2004a,b). But in the case of imidazolium-based SAILs, the estimation of critical micelle concentration using this method can be carried out preferably because these ILs have imidazolium rings present in their structure. Due to the presence of these rings, these SAILs can absorb in the entire UV-visible region (200-800 nm) and thus the estimation of critical micelle concentration using this method does not require any organic probe in their solution. The plot of absorbance vs. concentration of SAIL usually has two straight lines having a sharp point of intersection as represented in Figure 6D. This point of intersection in the plot is representative of the onset of micellization and the concentration of surface-active molecules corresponding to this point of intersection gives the value of CMC (Rather et al., 2015; Adil et al., 2016).

#### Other Techniques

The aggregation of surface-active molecules can also be further investigated by employing other techniques such as refractive index measurement, FTIR spectroscopy, dynamic light scattering (DLS), and many more. The refractive index measurement can also be utilized to determine the value of critical micelle concentration as the rate of change of refractive index of solution with the addition of surfactant is also varied upon micellization of surfactant monomers in solution (Tan et al., 2010; Shakeel et al., 2015). Also, FTIR spectroscopic technique can be employed to know about the structural alterations occurring in the solution with the micelle formation (Mehta et al., 2007; Pal et al., 2013; Singh and Kang, 2015; Banjare et al., 2017). Dynamic light scattering (DLS) can be employed to determine the size of micelles formed (Mata et al., 2004; Rai et al., 2010; Bhatt et al., 2014; Singh and Kang, 2015; Panda et al., 2018). Other techniques such as proton nuclear magnetic resonance (^1^H-NMR) spectroscopy, scanning electron microscopy (SEM) as well as tunneling electron microscopy (TEM) can also be employed to investigate the aggregation behavior of surface-active molecules (Inoue and Yamakawa, 2011; Mahajan et al., 2012; Bharmoria et al., 2013; Chabba et al., 2015; Pal and Punia, 2019a).

## Various Theoretical Models

The conventional surfactants as well as SAILs form micelles upon self-aggregation. On mixing two different kinds of surface-active molecules, there may be the formation of mixed micelles, which are more thermodynamically stable than simple micelles. Thus, the mixed micellization behavior of various surfactant mixtures is analyzed by utilizing various theoretical models. This leads to the determination of mixed micellar parameters which further can be utilized to know about the interactions prevailing in the mixed systems. Various theoretical models such as Clint, Rubingh, Motomura, and Rodenas are utilized by various researchers to evaluate mixed micellar parameters as well as ideal or non-ideal nature of mixing (Clint, 1975; Rodenas et al., 1999; Kabir-ud et al., 2010; Kumar and Rub, 2017; Azum et al., 2018; Das et al., 2018; Hoque et al., 2018; Kumar et al., 2018a; Pal and Punia, 2019a). In the case of mixed surfactant systems, the determination of the molar fraction of each surfactant in the aggregate iss difficult, which is needed to interpret the behavior of surfactant mixtures by using Clint relation as well as regular solution theory (RST). The equations and interpretations get varied according to the molar fraction definition (Letellier et al., 2011; Poša, 2014, 2019). In the case of ideal mixing, there is no net interaction between the components of the mixed system. The *cmc* of mixed systems in an ideal state can be evaluated by using Clint equation shown below.

(16) $$\begin{array}{l} \frac{1}{cmc^{*}}=\frac{\alpha_{1}}{cmc_{1}}+\frac{\left(1-\alpha_{1}\right)}{cmc_{2}} \end{array}$$

where cmc^*^ is the *cmc* of the mixed system in an ideal state, *cmc*_1_ and *cmc*_2_ represents critical micelle concentration of pure component 1 and 2, respectively, α_1_ denotes the mole fraction of component 1, (1 − α_1_) represents the mole fraction of component 2. If the values of cmc^*^ of mixed systems come out to be different than experimental *cmc* of that system, it signifies some sort of interactions is occurring within the mixed system. The less value of cmc^*^ as compared to experimental *cmc* (cmc^*^ < *cmc*) indicates positive deviation (antagonism) while the more value of cmc^*^ as compared to experimental *cmc* (cmc^*^ > *cmc*) indicates negative deviation (synergism) in the mixed system (Clint, 1975; Motomura et al., 1984; Rodenas et al., 1999; Kabir-ud et al., 2010; Kumar and Rub, 2017; Azum et al., 2018; Das et al., 2018; Hoque et al., 2018; Kumar et al., 2018a; Pal and Punia, 2019a).

Rubingh model can be further utilized to determine the micellar mole fraction $X_{1}^{Rub}$ and interaction parameter (β) by using the following equations (17) and (18).

(17) $$\begin{array}{l} \frac{{\left(X_{1}^{Rub}\right)}^{2}\ln\left[\left(\alpha_{1}cmc/X_{1}^{Rub}{cmc}_{1}\right)\right]}{{\left(1-X_{1}^{Rub}\right)}^{2}\ln\left[\left({1-\alpha }_{1}\right)cmc/\left(1-X_{1}^{Rub}\right){cmc}_{2}\right]}=1 \end{array}$$

(18) $$\begin{array}{l} \beta =\frac{\ln\;\left(\frac{\alpha_{1}cmc}{X_{1}^{Rub}{cmc}_{1}}\right)}{{\left(1-X_{1}^{Rub}\right)}^{2}} \end{array}$$

The value of β tells us about the type of interactions present between the components of the mixed system. If the value of β is found to be negative, it indicates synergistic interactions whereas if it comes out to be positive, it specifies antagonistic interactions between the components of the system. The zero value obtained for β indicates the ideal mixing of components in which no interactions are prevailing among components of the system (Paria, 2006).

Motomura model can also be utilized to determine the micellar mole fraction of constituent 1 ($X_{1}^{M}$), by using the following equations.

(19) $$\begin{array}{l} X_{1}^{M}=\bar{\alpha_{1}}-\frac{\left(\bar{\alpha_{1}}\bar{\alpha_{2}}/\bar{cmc}\right){\left(\partial \bar{cmc}/\partial \bar{\alpha_{1}}\right)}_{T,P}}{1-\frac{\delta \upsilon_{1,c}.\upsilon_{2,d}}{\upsilon_{1,c}.\upsilon_{2}\bar{\alpha_{1}}+\upsilon_{2,d}.\upsilon_{1}\bar{\alpha_{2}}}} \end{array}$$

(20) $$\begin{array}{l} \bar{cmc}=\left(\upsilon_{1}\alpha_{1}+\upsilon_{2}\alpha_{2}\right)cmc \end{array}$$

(21) $$\begin{array}{l} \alpha_{i}=\frac{\upsilon_{i}\alpha_{i}}{\upsilon_{1}\alpha_{1}+\upsilon_{2}\alpha_{2}}\;\left(i=1,2\right) \end{array}$$

where υ_1,*c*_ stands for component 1 breaking down into a-ions and c- ions, υ_2,*d*_ denotes for component 2 dissociating into b-ions and d-ions. The c- and d-ions are the counter ions of individual components, $\bar{\alpha_{i}}$ denotes the bulk mole fraction, υ_*i*_ represents the number of ions released from i^th^ component, δ expresses the Kronecker delta which is equal to one for similar counterions and zero for dissimilar counterions (Clint, 1975; Motomura et al., 1984; Rodenas et al., 1999; Kabir-ud et al., 2010; Kumar and Rub, 2017; Azum et al., 2018; Das et al., 2018; Hoque et al., 2018; Kumar et al., 2018a; Pal and Punia, 2019a).

Similarly, the Rodenas model can also be used to calculate the micellar mole fraction of component 1 ($X_{1}^{Rod}$) by using the following equation.

(22) $$\begin{array}{l} X_{1}^{Rod}=-(1-\alpha_{1})\alpha_{1}\frac{d\ln cmc}{d\alpha_{1}}+\alpha_{1} \end{array}$$

To evaluate the micellar mole fraction of component 1 ($X_{1}^{id}$) in an ideal state, the following equation can be utilized.

(23) $$\begin{array}{l} X_{1}^{id}=\frac{\alpha_{1}{cmc}_{2}}{\alpha_{1}{cmc}_{2}\;+\;\alpha_{2}{cmc}_{1}} \end{array}$$

The magnitude of $X_{1}^{id}/X_{1}^{Rub}/X_{1}^{M}/X_{1}^{Rod}$ tell us about the contribution of both the components toward formed mixed micelles. The activity coefficients of component 1 ($f_{1}^{Rub}$) and component 2 ($f_{2}^{Rub}$) in the case of the Rubingh model can be calculated using equations (24) and (25).

(24) $$\begin{array}{l} f_{1}^{Rub}=\exp\left[\beta \;{\left(1-X_{1}^{Rub}\right)}^{2}\right] \end{array}$$

(25) $$\begin{array}{l} f_{2}^{Rub}=\exp\left[\beta \;{\left(X_{1}^{Rub}\right)}^{2}\right] \end{array}$$

Whereas, in the case of Motomura and Rodenas models, these can be obtained by employing equations (26) and (27).

(26) $$\begin{array}{l} f_{1}^{a}=\left(\alpha_{1}\;cmc\right)/\left(X_{1}^{a}{cmc}_{1}\right) \end{array}$$

(27) $$\begin{array}{l} f_{2}^{a}=\left(1-\alpha_{1}\right)cmc/\left(1-X_{1}^{a}\right){cmc}_{2} \end{array}$$

where *a* in superscript stands for both Motomura and Rodenas. The values of activity coefficients can be used to calculate the excess free energy of mixed micellization${\Delta G}_{ex}^{a}$ by using the following equation (28).

(28) $$\begin{array}{l} \Delta G_{ex}^{a}=RT\{X_{1}^{a}\ln f_{1}^{a}+\left(1-X_{1}^{a}\right)\ln f_{2}^{a} \end{array}$$

where ${\Delta G}_{ex}^{a}$ symbolize ${\Delta G}_{ex}^{Rub}/{\Delta G}_{m}^{M}/{\Delta G}_{m}^{Rod}$ based upon Rubingh, Motomura, and Rodenas models. The magnitude of ${\Delta G}_{ex}^{Rub}/{\Delta G}_{m}^{M}/{\Delta G}_{m}^{Rod}$ tell us about the stability of formed mixed micelles (Clint, 1975; Motomura et al., 1984; Rodenas et al., 1999; Kabir-ud et al., 2010; Kumar and Rub, 2017; Azum et al., 2018; Das et al., 2018; Hoque et al., 2018; Kumar et al., 2018a; Pal and Punia, 2019a).

## Self-Assembly of Sails

SAILs can be categorized into various types depending upon their structural features. They have the unique property of self-assembling into various shapes such as micelles, vesicles, bilayer membranes, etc. They possess the properties of ILs as well as surfactants and show more tendency of micellization as compared to traditional surfactants. Depending upon their self-assembly behavior, they can be utilized in a variety of fields and can replace many conventional surfactants. Thus, the self-assembly of various SAILs i.e., anionic surface-active ionic liquids (ASAILs), cationic surface-active ionic liquids (CSAILs), Gemini cationic surface-active ionic liquids (GCSAILs), dicationic surface-active ionic liquids (DCSAILs) as well as catanionic surface-active ionic liquids (CASAILs) have been investigated by many researchers.

Sun et al. (2019) synthesized four dodecyl sulfate-based anionic SAILs and characterized them by utilizing ^1^H-NMR spectroscopy. Then various physicochemical parameters and thermodynamic parameters were evaluated using surface tension measurement and conductivity measurements. Also, density functional theory is applied to understand the interactions prevailing in gelatin and anionic SAILs. Qin et al. (2020) synthesized 1-butyl-3-ethoxy carbonyl imidazolium dodecanesulfate ([Etbim]DS) and analyzed its micellization behavior by applying surface tension, steady-state fluorescence spectroscopy, electrical conductivity measurements, and density functional theory (DFT). Li et al. (2017) synthesized 3-methyl-(ethoxycarbonylmethyl)-imidazolium dodecylsulfate ([Etmim]DS), which was found to be better than 3-methyl-(ethoxycarbonylmethyl)-imidazolium chloride [Etmim]Cl for white hide powder dissolution and characterized its structure by utilizing 1H NMR. Further various physicochemical performances of white hide powder/[Etmim]DS were evaluated by employing various techniques such as tensiometry, electrical conductivity, and fluorimetry, and the regenerated white hide powder was characterized by using FT-IR, TGA, DSC, and XRD techniques. Rao et al. (2012) synthesized biamphiphilic ionic liquids (BAILs) i.e., ([C_n_H_2n+1_mim][C_m_H2_m+1_OSO_3_];*n* = 4, 6, or 8; m = 8, 12) and studied their self-assembly behavior by using various techniques highlighting their application in the preparation of nanomaterials.

Cheng et al. (2014) investigated the aggregation behavior of AOT- and DEHP-based anionic SAILs by employing surface tension measurements, conductivity measurements, fluorescence spectroscopy as well as dynamic light scattering (DLS) measurements and concluded that 1-butyl-3-methylimidazolium bis(2-ethylhexyl) sulfosuccinate (C_4_mim-AOT) and 1-butyl-3-methylimidazolium bis(2-ethylhexyl) phosphate (C_4_mim-DEHP) are more super active than their sodium analogs.

Brown et al. (2012) synthesized three anionic SAILs with 1-butyl-3-methyl-imidazolium cations i.e., bmim DS, bmim AOT, and bmim TC. Various physicochemical properties were evaluated by utilizing various techniques such as density, viscosity, and surface tension measurements highlighting their applications in various fields.

Jiao et al. (2013) investigated the aggregation behavior of a series of salt-free catanionic SAILs by employing surface tension, fluorescence, and electrical conductivity measurements. Pal and Maan (2019) studied the interactions prevailing among 1-butyl-3-methylimidazolium dodecyl benzenesulfonate [C_4_mim][DBS] and non-ionic polymer polyethylene glycol 400 (PEG 400) in aqueous solution by employing tensiometry, conductivity, dynamic light scattering (DLS), nephelometric turbidity, and rheology.

Pal and Saini (2020) synthesized and characterized SAILs having 1-alkyl-3-methylimidazolium cationic moiety and dodecyl benzenesulfonate-based anionic moiety i.e., [C_5_mim][DBS] and [C_7_mim][DBS]. They investigated their aggregation behavior by using conductivity, surface tension measurements, UV-visible and fluorescence spectroscopic techniques, and determined their value of CMC, thermodynamic parameters as well as surface-active parameters. Garcia et al. (2020) investigated the aggregation behavior and antimicrobial activities of catanionic mixtures prepared by mixing non-functionalized, ester or amide functionalized methylimidazolium or pyridinium-based surface-active ionic liquids (SAIL-Br) and sodium bis(2-ethyl-1-hexyl) sulfosuccinate (Na-AOT) in equimolar concentrations. The elongation of the hydrophobic tail of ionic liquid leads to a lower value of critical aggregation concentration (CAC). The high surface activity, less value of critical aggregation concentration (CAC), high antimicrobial activity, and low toxicity has been observed in the case of the prepared catanionic mixtures.

Vaghela et al. (2011) determined the self-aggregation behavior of ILs [C_n_mim][X] where *n* = 4, 6, 8, and [X] = Cl^−^, Br^−^ and I^−^ by using various techniques and evaluated various surface-active and thermodynamic parameters of micellization. Singh and Kumar (2007) investigated the self-assembly of imidazolium-based ILs [C_4_mim][Cl], [C_8_mim][Cl], [C_4_mim][BF_4_] and [C_4_mpy][Cl] by using ^1^H NMR, refractive index, and fluorescence spectroscopy and determined the value of CMC.

Singh and Kumar (2008b) studied the aggregation behavior of [C_4_mim][BF_4_], [C_8_mim][BF_4_], [C_8_mim][Cl] and [C_4_mpy][Cl] in aqueous solution at 298.15 K by using conductivity measurement, density, and speed of sound measurements and determined various micellization parameters. Wang et al. (2008) studied the aggregation of 1-alkyl-3-methylimidazolium salts [C_8_mim], where X = Cl^−^, Br^−^, [NO_3_]^−^, [CH_3_COO]^−^, [CF_3_COO^−^, [CF_3_SO_3_]^−^, and [ClO_4_]^−^), 1-octyl-4-methyl pyridinium bromide ([C_8_pyr]Br), and 1-methyl-1-octylpyrrolidinium ([C_8_mpyrr]Br) by conductivity, volume, fluorescence, dynamic light scattering, and transmission electron microscopy in aqueous solution. El Seoud et al. (2007) synthesized ILs based upon 1-alkyl-3-methylimidazolium chloride RMeImCl where *R* = C_10_, C_12_, C_14_, and C_16_, respectively, and investigated their aggregation in an aqueous medium by using surface tension, conductivity measurement, fluorescence spectroscopy as well as static light scattering technique. It is observed that the increase in length of the alkyl chain led to a decrease in value of CMC, an increase in the aggregation number as well as a decrease in the minimum area of the head group at the air/water interface.

Vanyúr et al. (2007) studied the micelle formation of 1-alkyl-3-methylimidazolium bromide (C_n_MImBr) in an aqueous solution at 298 K using conductivity as well as fluorescence spectroscopy. Their CMC values were found to be between that of alkyl trimethylammonium bromides (C_n_TAB) and sodium alkyl sulfates having the same alkyl chain length. Łuczak et al. (2010) tells that the antimicrobial activity of ILs depends upon the length of the alkyl chain and the nature of anion present in their structure by investigating the aggregation behavior and antimicrobial activity of 30 compounds. The presence of a longer alkyl chain in ionic liquid leads to a lower value of minimal inhibitory concentration (MIC) showing that the increase in chain length led to better antifungal and antibacterial activities. Dong et al. (2007, 2008) investigated the self-assembly behavior of C_12_mimBF_4_, C_12_mimBr, C_14_mimBr, and C_16_mimBr by using surface tension and steady-state fluorescence measurements at 298 K in an aqueous medium as well as in presence of sodium halide salts i.e., NaCl, NaBr, and NaI. The decrease in value of CMC has been observed with the addition of salts as in the case of conventional ionic surfactants. Shi et al. (2011) investigated the micellization of N-aryl imidazolium ILs [C_n_pim]Br (*n* = 10, 12, and 14), in an aqueous solution by various techniques and observed that these ILs have a low value of CMC as compared to 1,3-dialkyl imidazolium ILs [C_n_mim]Br] due to enhanced π-π interactions among the adjacent 2,4,6-trimethylphenyl groups leading to more tendency of micellization.

Li et al. (2010) synthesized chiral long-chain ionic liquid S-3-hexadecyl-1-(1-hydroxy-propan-2-yl)-imidazolium bromide ([C_16_hpim]Br), which is found to have more tendency of micelle formation as compared to conventional surfactants. Singh et al. (2016) synthesized ILs [C_16_mim][HBS], [C_16_mim][BS] and [C_16_mim][PTS], where [HBS] corresponds to 4-hydroxybenzenesulfonate, [BS] denotes benzenesulfonate and [PTS] signifies p-toluenesulfonate. Their aggregation and surface-active nature were investigated by using surface tension, conductivity, and fluorescence measurements. Their CMC is found to be less than that of corresponding ILs as well as surfactants with non-aromatic counterions.

Bhadani and Singh (2011) synthesized twelve Gemini surfactants having C_12_, C_14_, C_16_, and C_18_ chain lengths having different spacers and determine their aggregation behavior and DNA binding affinity using conductivity, surface tension measurements, agarose gel electrophoresis, and ethidium bromide exclusion experiments. Ao et al. (2008, 2009) synthesized ionic liquid-type Gemini imidazolium surfactants i.e., ([C_12_− s–C_12_im]Br_2_, s = 2,4,6) and ([C_n_−4−C_n_im]Br_2_, *n* = 10, 12, 14) and their aggregation in an aqueous medium was investigated by using electrical conductivity and surface tension measurements leading to the determination of various surface-active and thermodynamic parameters of micellization. Also, the effect of the addition of halide salts on the aggregation behavior of these Gemini ILs was evaluated. Shaheen et al. (2020) synthesized three Gemini surfactants, 1, 1′- (propane-1,3-diyl-2-ol) bis(3-alkyl-1H- imidazol-3-ium)bromide, [C_n_Im-3OH-ImC_n_]Br_2_, where *n* = 12,14,16 and studied their micellization using various techniques. Then their cytotoxic properties were analyzed highlighting their use in industrial and biomedical fields.

**Figure 7:**
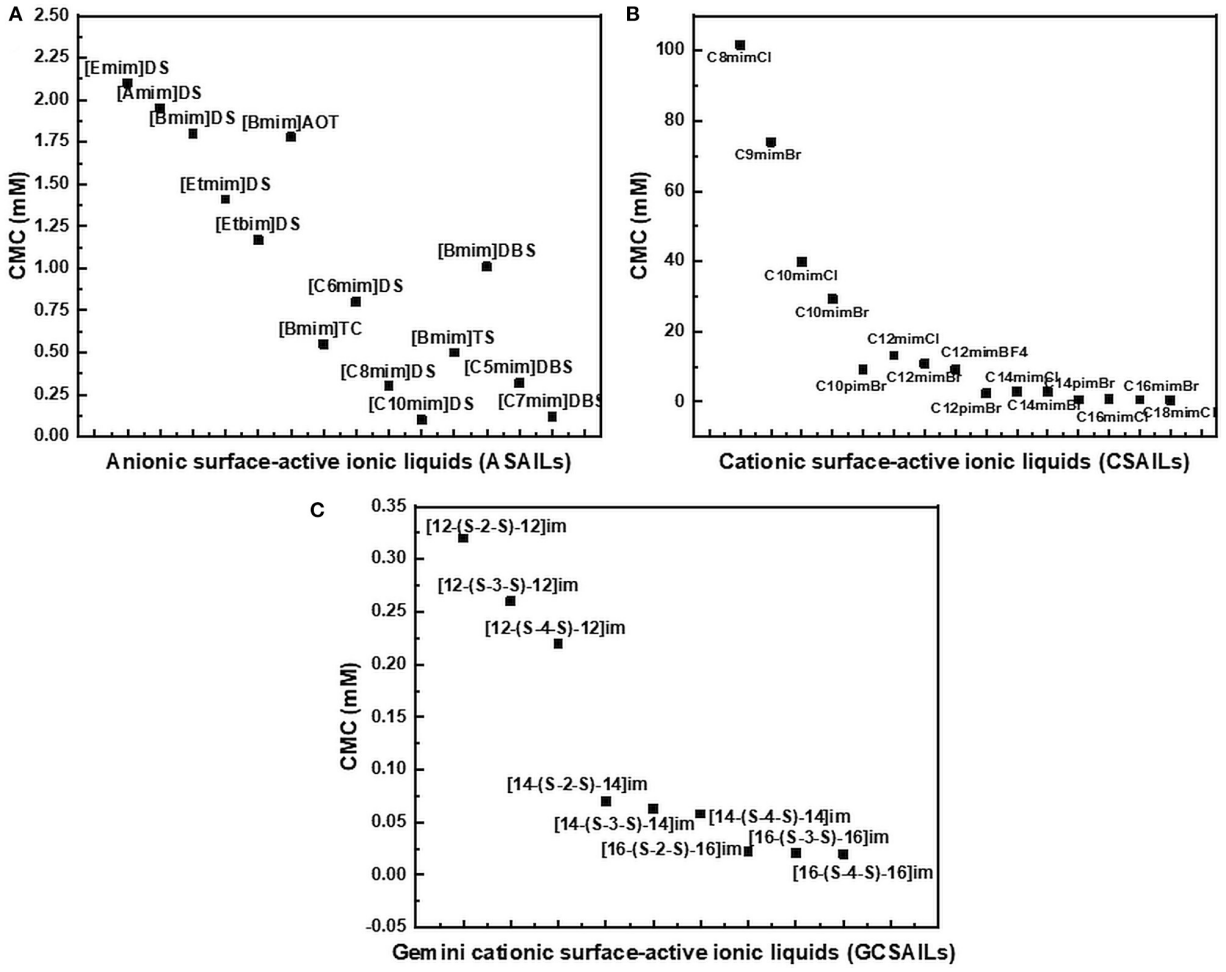
Plot of CMC values of various **(A)** anionic surface-active ionic liquids (ASAILs) **(B)** cationic surface-active ionic liquids (CSAILs) **(C)** Gemini cationic surface-active ionic liquids (GCSAILs).

**Figure 8:**
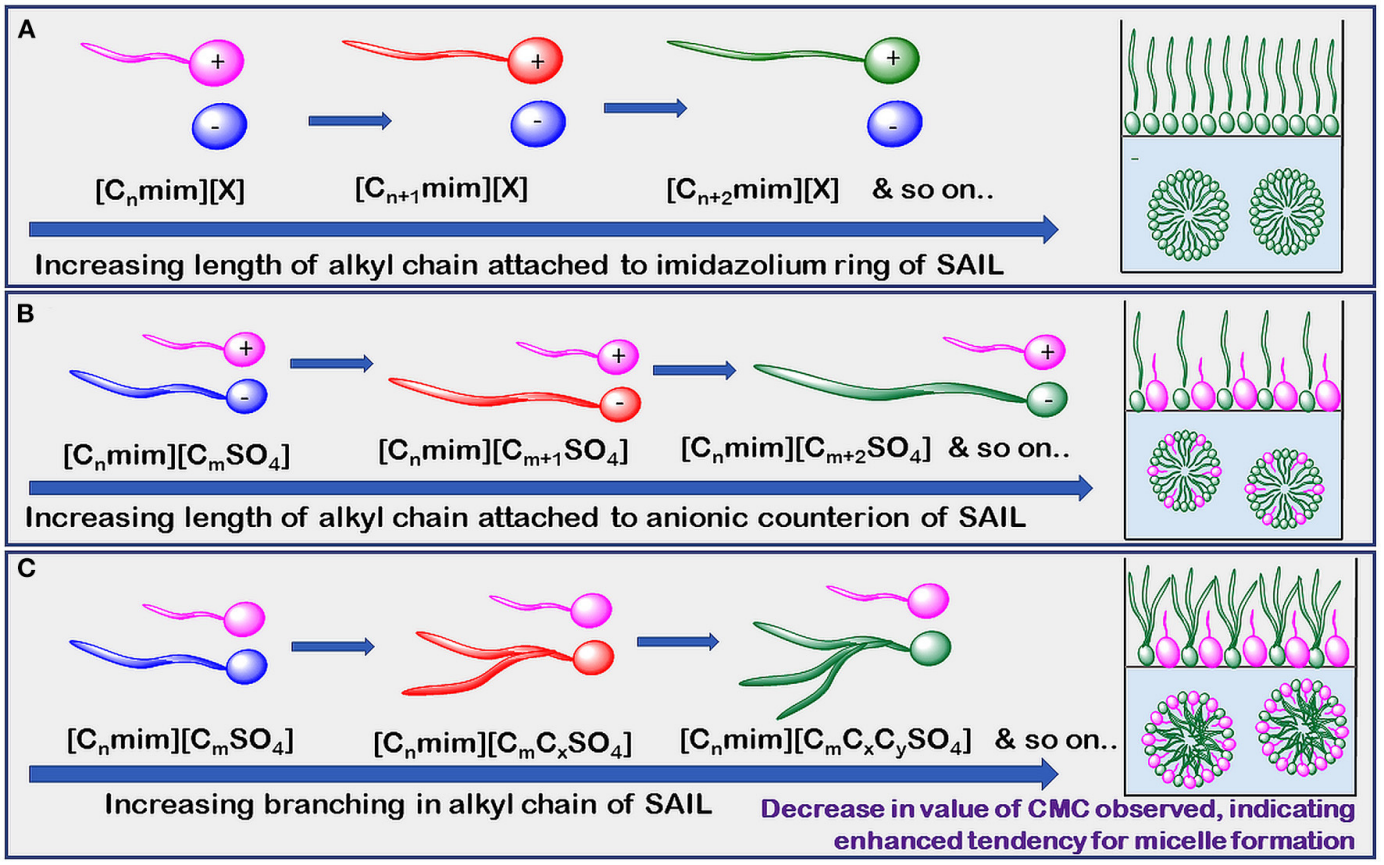
Schematic diagram of CMC (mM) variation by increasing length of alkyl chain attached to **(A)** imidazolium ring of SAIL **(B)** anionic counterion of SAIL and **(C)** branching of SAILs.

The reported values of CMC, the surface tension at CMC (γCMC), surface excess concentration (Γmax), and minimum area per molecule at air-water interface (*A*min) for various synthesized ILs have been presented in Tables 1–3. The values of CMC of various SAILs have been plotted in Figure 7. It can be well-observed from the figure that the value of CMC is found to be more in the case of the presence of a short alkyl chain in the structure of IL, whereas it is less in the case of ILs with longer alkyl chains present in their structure. In the case of ILs having the same counterion, the value of CMC decreases with the increase in length of hydrophobic alkyl chain present in the structure of ILs. It means acceleration in the tendency of micelle formation has been observed with the increase in length of alkyl chain present in the structure of surface-active agent. In case of micelle formation, the hydrophilic head groups repel each other electrostatically, whereas hydrocarbon alkyl chains attract each other by hydrophobic force. The observed decrement in value of CMC on increasing alkyl chain length can be explained by the fact that there exist favorable interactions between long hydrophobic alkyl chains of surface-active molecule disrupting the structure of solvent, thereby increasing the free energy of system, which is the driving force behind the micellization process (Rosen and Kunjappu, 2012). In case of GCSAILs having thioether spacer, the decrease in value of CMC with the increase in spacer length has been observed as shown in Figure 7. This may be due to the interaction of sulfur atom of spacer forming H-bonds with water, thereby reducing unfavorable hydrocarbon-water contacts helping to orient the spacer at micelle water interface. Also, coulombic repulsions between headgroups decrease with the additional hydration of the hydrophilic spacer chain, thereby enhancing the aggregation at lower concentrations leading to a decrease in the value of CMC. But in the case of increasing length of hydrophobic spacer group in GCSAILs, the value of CMC increases as observed from Table 3. It may be due to the difficulty in incorporating the longer hydrophobic moiety in the hydrophilic micellar outer region causing steric inhibition of intramolecular chain-chain association, thereby retarding the aggregation tendency of GCSAILs with the increase in length of hydrophobic spacer group (Wang et al., 2004; Han and Wang, 2011). Figure 8 represents the schematic diagram for variation in the value of CMC with the increase in the length and branching of alkyl chain attached to imidazolium ring or counterion of SAIL.

**Table 1:** Reported values of critical micelle concentration (CMC), the surface tension at CMC (γCMC), surface excess concentration (Γmax), and minimum area per molecule at air–water interface (*A*min) for anionic surface-active ionic liquids (ASAILs) and catanionic surface-active ionic liquids (CASAILs) at 298.15 K in aqueous medium.

**SAILs**	***CMC* (mM)**	**γCMC (mN.m^**−1**^)**	**Γmax (μmol.m^**−2**^)**	***A*min(Å^**2**^)**	**References**
[Bmim]AOT	1.78	25.7	—	86	Brown et al., 2012
	1.70	26.1	1.47	113	Cheng et al., 2014
[C4mim]DEHP	9.3	25.5	1.41	118	Cheng et al., 2014
[Bmim]TC	0.55	24.8	—	111	Brown et al., 2012
[Emim]DS	2.10	38.97	2.49	66.58	Sun et al., 2019
[Amim]DS	1.95	38.31	2.54	65.42	Sun et al., 2019
[Bmim]DS	1.80	31.9	2.53	66.0	Qin et al., 2020
	2.30	32.9	—	56.0	Brown et al., 2012
	2.4	34.4	2.4	67.8	Rao et al., 2012
	0.95	37.9	2.23	74.36	Sun et al., 2019
[Etmim]DS	1.41	27.2	4.38	38.0	Li et al., 2017
	0.86	29.26	2.21	75.27	Sun et al., 2019
[Etbim]DS	1.17	33.97	2.21	75.14	Qin et al., 2020
[C_6_mim]DS	0.8	30.0	2.08	80	Jiao et al., 2013a
	1.1	27.1	2.4	68.5	Rao et al., 2012
[C_8_mim]DS	0.3	26.9	2.33	71	Jiao et al., 2013a
	0.4	26.0	2.4	68.5	Rao et al., 2012
[C_10_mim]DS	0.1	25.4	2.36	70	Jiao et al., 2013a
[C4mim]OS	34.9	26.1	1.9	87.1	Rao et al., 2012
[C6mim]OS	14.2	25.6	1.9	84.7	Rao et al., 2012
[C8mim]OS	4.1	24.4	2.5	66	Rao et al., 2012
[C_4_mim][C_10_SO_4_]	8.8	34.7	2.81	59	Jiao et al., 2013a
[C4mim][ C_14_SO_4_]	0.5	30.5	1.66	10	Jiao et al., 2013a
[C_4_mim]DBS	1.01	36.00	3.22	51.50	Pal and Maan, 2019
[C5mim]DBS	0.32	30.92	1.91	86.76	Pal and Saini, 2020
[C7mim]DBS	0.12	34.21	1.35	122.64	Pal and Saini, 2020
C_8_MeIm-AOT	0.21	26.0	1.7	100	Garcia et al., 2020
C_10_MeIm-AOT	0.06	26.3	1.8	90	Garcia et al., 2020
C_12_MeIm-AOT	0.021	26.6	2.1	80	Garcia et al., 2020
C_14_MeIm-AOT	0.007	29.0	2.2	76	Garcia et al., 2020

**Table 2:** Reported values of critical micelle concentration (CMC), the surface tension at CMC (γCMC), surface excess concentration (Γmax), and minimum area per molecule at air–water interface (*A*min) for cationic surface-active ionic liquids (CSAILs) at 298.15 K in aqueous medium.

**CSAILs**	***CMC* (mM)**	**γCMC (mN.m^**–1**^)**	**Γmax (μmol.m^**–2**^)**	***A*min (Å^**2**^)**	**References**
[C4mim]Cl	935^*b*^	—	—	—	Singh and Kumar, 2007
[C4mim]BF4	952^*b*^	—	—	—	Singh and Kumar, 2007
	729^*c*^	—	—	—	Singh and Kumar, 2008b
[C6mim][Cl]	902.8^*a*^	39.2	1.3	130	Vaghela et al., 2011
[C6mim][Br]	470^*a*^	33.6	2.0	83	Vaghela et al., 2011
[C6mim][I]	250.3^*a*^	34	1.3	124	Vaghela et al., 2011
[C8mim]Cl	101.7^*a*^	28.3	1.6	104	Vaghela et al., 2011
	90^*b*^	—	—	—	Singh and Kumar, 2007
	100^*c*^	—	—	—	Singh and Kumar, 2008b
[C8mim]Br	120^*a*^	28.7	2.7	60	Vaghela et al., 2011
[C8mim]I	94.9^*a*^	28.2	1.4	117	Vaghela et al., 2011
[C8mim]BF4	28^*c*^	—	—	—	Singh and Kumar, 2008b
[C8mim][CH3COO]	220^*c*^	—	—	—	Wang et al., 2008
[C8mim][NO3]	150^*c*^	—	—	—	Wang et al., 2008
[C8mim][CF3COO]	120^*c*^	—	—	—	Wang et al., 2008
[C9mim]Br	74^*c*^	—	—	—	Vanyúr et al., 2007
[C10mim]Cl	39.9	—	—	85	El Seoud et al., 2007
[C10mim]Br	29.3^*a*^	39.7	1.72	96.7	Dong et al., 2007
	41^*c*^	—	—	—	Vanyúr et al., 2007
[C10pim]Br	9.30^*a*^	38.3	1.90	87.4	Shi et al., 2011
[C12mim]Cl	13.17^*a*^	—	—	72	El Seoud et al., 2007
[C12mim]Br	10.9	39.4	1.91	86.8	Dong et al., 2008
	9.8^*c*^	—	—	—	Vanyúr et al., 2007
[C12pim]Br	2.34^*a*^	38.4	2.09	79.4	Shi et al., 2011
[C12mim]BF4	9.2 ^*a*^	38.2	2.16	76.7	Dong et al., 2007
[C14mim]Cl	2.98^*a*^	—	—	56	El Seoud et al., 2007
	3.2^*a*^	—	—	—	Łuczak et al., 2010
[C14mim]Br	2.8^*a*^	39.2	1.96	84.7	Dong et al., 2008
	2.5^*c*^	—	—	—	Vanyúr et al., 2007
[C14pim]Br	0.61^*a*^	38.3	2.21	75.1	Shi et al., 2011
[C16mim]Cl	0.87^*a*^	—	—	49	El Seoud et al., 2007
	1.1^*c*^	—	—	—	Łuczak et al., 2010
[C16mim]Br	0.55^*a*^	39.1	2.03	81.6	Dong et al., 2008
	0.6^*c*^ 1	—	—	—	Vanyúr et al., 2007
[C16hpim]Br	0.56^*a*^	43.4	1.99	83.2	Li et al., 2010
[C16mim][HBS]	0.38^*a*^	33.5	2.62	63	Singh et al., 2016
[C16mim][BS]	0.33^*a*^	33.7	2.26	73	Singh et al., 2016
[C16mim][PTS]	0.20^*a*^	31.0	2.13	78	Singh et al., 2016
[C18mim]Cl	0.45^*a*^	—	—	—	Łuczak et al., 2010

a *Surface tension measurement*.

b *NMR spectroscopy*.

c *Conductivity measurement*.

**Table 3:** Reported values of critical micelle concentration (CMC), the surface tension at CMC (γCMC), surface excess concentration (Γmax), and minimum area per molecule at air–water interface (*A*min) for Gemini cationic surface-active ionic liquids (GCSAILs) at 298.15 K in aqueous medium.

**GCSAILs**	***CMC* (mM)**	**γ_**CMC**_ (mN.m^**–1**^)**	**Γmax (μmol.m^**–2**^)**	***A*min (Å^**2**^)**	**References**
[12-(S-2-S)-12]im	0.32	39.7	2.60	63	Bhadani and Singh, 2011
[12-(S-3-S)-12]im	0.26	40.7	2.13	77	Bhadani and Singh, 2011
[12-(S-4-S)-12]im	0.22	40.8	2.06	80	Bhadani and Singh, 2011
[14-(S-2-S)-14]im	0.07	42.9	2.12	78	Bhadani and Singh, 2011
[14-(S-3-S)-14]im	0.063	45.8	3.09	53	Bhadani and Singh, 2011
[14-(S-4-S)-14]im	0.058	46.6	3.1	53	Bhadani and Singh, 2011
[16-(S-2-S)-16]im	0.022^*a*^	—	—	—	Bhadani and Singh, 2011
[16-(S-3-S)-16]im	0.021^*a*^	—	—	—	Bhadani and Singh, 2011
[16-(S-4-S)-16]im	0.020^*a*^	—	—	—	Bhadani and Singh, 2011
[C12–2–C12im]Br2	0.55	33.6	1.23	135	Ao et al., 2009
[C12–4–C12im]Br2	0.72	35.7	1.19	140	Ao et al., 2009
[C12–6–C12im]Br2	0.78	39.5	1.16	143	Ao et al., 2009
[C10-4-C10im]Br2	4.5	35.2	1.25	133	Ao et al., 2008
[C12-4-C12im]Br2	0.72	35.7	1.19	140	Ao et al., 2008
[C14-4-C14im]Br2	0.10	37.2	0.88	188	Ao et al., 2008
[C_12_Im-3OH-C_12_Im]Br_2_	0.057	—	—	—	Shaheen et al., 2020
[C_14_Im-3OH-C_14_Im]Br_2_	0.036	—	—	—	Shaheen et al., 2020
[C_16_Im-3OH-C_16_Im]Br_2_	0.018	—	—	—	Shaheen et al., 2020

a *, Surface tension measurement*.

## Mixed Micellization Behavior

Apart from the self-assembly of various imidazolium-based ILs, their micellization in combination with surfactants has become the focus of much research. The mixing of ILs with conventional surfactants may lead to the formation of mixed micelles. The alterations induced in the aggregation behavior of SAIL or surfactant monomers with the addition of conventional surfactants or ILs are investigated by various researchers. There are a lot of ILs/surfactants, combinations, which can be utilized to analyze the mixed micellization behavior, physicochemical properties as well as various types of interactions present among them. This type of investigation may contribute to the utilization of these ILs/surfactants combinations in place of conventional surfactants with better applicability and performance.

### ILs/Surfactants Mixed Micellization

The modulation in the micellization behavior of various kinds of conventional surfactants has been observed in the presence of various concentrations of imidazolium-based ILs and concluded these ILs/surfactant systems can serve as better systems in many industries where surfactants are employed. Anionic surfactants can be regarded as an important category of surfactants having a wide variety of applications in the cleaner and detergent industry. Important anionic surfactants are those based upon alkyl sulfates or alkylbenzene sulfonates. Examples of commercially important anionic surfactants include sodium dodecyl sulfate (SDS), sodium dodecylbenzene sulfonate (SDBS), sodium lauryl ether sulfate (SLES), and sodium dioctyl sulfosuccinate (Na-AOT). Thus, the influence of adding various ILs on the aggregation of sodium dodecyl sulfate (SDS) has been observed to understand various types of interactions prevailing among the molecules.

Pal and Chaudhary (2013a,b, 2014) investigated the effect of adding different concentrations of various ILs such as 3-methyl-1-pentylimidazolium tetra-fluoroborate [C_5_mim][BF_4_], 3-methyl-1-pentylimidazolium hexafluorophosphate [C_5_mim][PF_6_], 3-methyl-1-pentylimidazolium bromide, [C_5_mim][Br] and 1-heptyl-3-methylimidazolium bromide, [C_7_mim][Br] on the aggregation behavior of sodium dodecyl sulfate (SDS) at various temperatures. Javadian et al. (2014) analyzed the modulation in the micellization behavior and surface activity of sodium dodecyl sulfate (SDS) with the addition of cationic ILs i.e., 1-butyl-3-methylimidazolium chloride (BMImCl), 1-butyl-3-methylimidazolium bromide (BMImBr), 1-hexyl-3-methyl-imidazolium chloride (HMImCl), and 1-hexyl-3-methyl-imidazolium bromide (HMImBr). This study highlights the decrease in value of CMC with the addition of ILs and the type of interactions present among the molecules as well as change in micellar phase from vesicles to micelles with the increase in the [SDS]/[IL] ratios.

Our group (Kumar and Kaur, 2020a,b) determines the influence of various concentrations of four imidazolium ILs with varying chain length such as 1-propyl-3-methylimidazolium bromide [C_3_mim][Br], 1-butyl-3-methylimidazolium bromide [C_4_mim][Br], 1-pentyl-3-methylimidazolium bromide [C_5_mim][Br] and 1-hexyl-3-methylimidazolium bromide [C_6_mim][Br] on the aggregation behavior of anionic surfactant sodium hexadecyl sulfate (SHS), in which increase in value of CMC has been observed with the addition of ILs, signifying the delay in micellization tendency by adding ILs in the system. However, this increase in value of CMC is more in case of the addition of IL having a short alkyl chain attached to it in comparison to that having a longer alkyl chain.

The influence of various ILs upon the micellization of various cationic surfactants has been investigated by Pal et al. (Chaudhary and Pal, 2014; Pal and Chaudhary, 2015; Pal and Pillania, 2015a,b, 2016b; Pal and Punia, 2019b). The alteration induced in the aggregation behavior of tetradecyltrimethylammonium bromide (TTAB) was investigated in the presence of ILs namely 1-butyl-2,3-dimethylimidazolium bromide [bdmim][Br], 1, 2-dimethyl-3-octylimidazolium chloride [odmim][Cl] and 3-methyl-1-pentylimidazolium hexafluorophosphate [C_5_mim][PF_6_]. The decrease in value of CMC of TTAB has been observed with the increase in the concentration of [bdmim][Br] and [odmim][Cl], whereas an increase in value of CMC is observed in the case of the addition of [C_5_mim][PF_6_]. Thus, the delay or better tendency of micellization of any surfactant with the addition of any IL depends upon the type of interactions among the molecules constituting the system. Thus, a detailed study of various facts observed from the experimental studies of various combinations can give better insight for the systems to have desired properties as well as micellization tendencies. Similarly, the addition of [C_5_mim][PF_6_] to the other cationic surfactants such as cetyltrimethylammonium bromide (CTAB) and cetyltrimethylammonium chloride (CTAC) give rise to the same result of the decrease in tendency of micellization. But the addition of [bdmim][Br] to CTAB lead to an increase in the tendency of micellization evident from the decrease in value of CMC of CTAB by an increase in the concentration of IL [bdmim][Br]. The same trend of increase in micellization tendency of surfactant has been observed in the case of addition of 1-butyl-2,3-dimethylimidazolium chloride, [bdmim][Cl] to cationic surfactant dodecyltrimethylammonium bromide (DTAB). Apart from evaluating CMC for various systems, they have also calculated various thermodynamic parameters of micellization.

Instead of cationic and anionic surfactants, other categories of surfactants such as non-ionic as well as zwitterionic surfactants are also investigated for the modification in their aggregation behavior with the ionic liquid addition. Inoue and Yamakawa (2011) analyzed the micellization behavior of polyoxyethylene-type non-ionic surfactants (C_n_E_m_) with varying chain length in 1-butyl-3-methylimidazolium tetrafluoroborate (bmimBF_4_). The linear decrease in the logarithmic value of CMC has been observed with the increase in length of hydrocarbon chain of surfactant similar to that observed in the case of the aqueous solution. Comelles et al. (2012) determines the interaction between an ethoxylated non-ionic surfactant (C_12−14_EO_8_) and three ILs (bmim-octyl SO_4_, bmim-methyl SO_4_, and bmim-BF_4_) in an aqueous solution. The study enables the appropriate selection of the mole fraction of surfactant for the particular application, depending upon the condition whether monomers or micellar aggregates are required to perform the function. Sun et al. (2014) studied the influence of methylimidazolium based ILs having hydrophobic anions on the aggregation of Triton X-114 and concluded that both the hydrophilic and hydrophobic ILs influence the micellization behavior of Triton X-114 evident from the change in the size of micellar aggregate, change in zeta potential, cloud point as well as CMC. Pal and Pillania (2016a, 2017) utilize 1-butyl-2,3-dimethylimidazolium bromide [bdmim][Br] and 1-hexyl-2,3-dimethylimidazolium bromide [hdmim][Br] to investigate alterations in micellization behavior of Triton X-100. They also utilize 1-butyl-2,3-dimethylimidazoliumbromide [bdmim][Br] and 1-butyl- 3-methylimidazolium bromide [bmim][Br] to investigate alterations micellization behavior of Triton X-45. The magnitude of CMC decreases at lower concentrations of IL, whereas it increases at higher concentrations of IL. Łuczak et al. (2015) investigated the alterations induced in the aggregation behavior of Tween 20 with the addition of eleven imidazolium-based ILs having different chain lengths as well as counter ions and highlights the fact that the tendency of self-assembly of surfactant is dependent upon the structures of both cationic as well as anionic species as well as the type of interactions prevailing among them. Pal et al. (2015) studies the effect of various concentrations of 3-methyl-1-pentylimidazolium tetrafluoroborate ([C_5_mim] [BF_4_]) on the aggregation of Tween 20 by employing fluorimetry and dynamic light scattering. The study indicates the decrease in tendency of micellization of Tween 20 by the addition of [C_5_mim] [BF_4_] as the value of CMC of surfactant increases and aggregation number decreases with the addition of ionic liquid.

Another category of surfactant namely zwitterionic surfactants is very little studied in the presence of ILs. However, Pal and Pillania (2019) determines the effect of addition of 1-butyl-2,3-dimethylimidazolium chloride [bdmim][Cl], 1-butyl-2,3-dimethylimidazolium bromide [bdmim][Br], 1-hexyl-2,3-dimethylimidazolium bromide [hdmim][Br], and 1-benzyl-2,3-dimethylimidazolium chloride [bzdmim][Cl] on the micellization and surface-active behavior of zwitterionic surfactant N-dodecyl-N,N-dimethyl- 3-ammonio-1-propanesulfonate (SB-12). The lowering in value of CMC of SB-12 has been observed with the addition of different concentrations of ILs. However, maximum lowering in CMC value has been achieved in the case of addition of [bzdmim][Cl].

### SAILs/Surfactants Mixed Micellization

Similar to ILs having a short alkyl chain attached to the imidazolium ring, the other ILs having longer alkyl chain present in their structure namely SAILs have also been utilized to investigate the interfacial, surface-active as well as mixed micellization behavior of their combinations with a variety of surfactants. Chabba et al. (2015) studies the interactions prevailing in the mixed system constituting 1-alkyl- 3-methylimidazolium chloride ([C_n_mim][Cl], where *n* = 8, 10, 12), and sodium dodecyl benzenesulfonate (SDBS) in aqueous media by employing various techniques at different mixing ratios of SDBS-SAIL mixtures. They highlighted the fact that the mixed systems are more surface-active as compared to individual ones. Further, the aqueous SDBS-SAIL mixtures in different mixing ratios have been utilized to prepare Ag nanoparticles and concluded that the morphology of prepared nanoparticles is dependent upon the composition of mixed micelles in the system.

Gu et al. (2015) determine the type of interactions in the mixed system of zwitterionic imidazolium-based SAILs N-alkyl-N′- carboxymethyl imidazolium inner salts ([N-C_n_,N′-CO_2_-Im], *n* = 12, 14), and dioctyl sulphosuccinate sodium salt, NaAOT in aqueous solution. The non-ideal behavior showing synergism, superior surface-activity, and stable micelle formation has been observed in the mixed system, highlighting its application in various industrial formulations.

Similar to anionic surfactants, the change in the micellization behavior of traditional cationic surfactants has also been examined in the presence of imidazolium-based SAIL molecules. Ali et al. (2016) investigated the modifications in the physicochemical properties of cationic surfactants cetylpyridinium chloride (CPC) and cetylpyridinium bromide (CPB) in the presence of 1-decyl-3-methylimidazolium chloride [C_10_mim][Cl]. The interactions in the mixed systems were found to be synergic as well as non-ideal with the dominance of hydrophobic interactions prevailing within the components of the mixed system. Qin and Wang (2017) determines the effect of temperature as well as the concentration of various mixtures of cetyltrimethylammonium bromide (CTAB) with SAILs (C_n_mimBr, *n* = 10, 12, 16) on various micellization as well as surface-active parameters and concluded the fact that the CMC values of mixed systems are very well-dependent upon CTAB concentration as well as the temperature of the system. The value of CMC decreased with an increase in the concentration of CTAB whereas it gets increased with the increase in temperature. Pal and Punia (2019b) evaluated various mixed micellar parameters as well as thermodynamic parameters of micellization for the mixtures of trisubstituted SAILs 1-tetradecyl-2,3-dimethylimidazolium bromide [C_14_bmim][Br] and cetyltrimethylammonium bromide (CTAB) in the absence and the presence of inorganic salt, sodium bromide (NaBr) in aqueous media using Rubingh, Clint and Motomura theory. The steady-state fluorescence quenching has been utilized to determine the aggregation number of the formed mixed micelle.

Thakkar et al. (2016) synthesized biamphiphilic SAILs 1-alkyl-3-methylimidazolium octylsulphates (C_n_mim C_8_SO_4_, *n* = 4, 6, 8, 10) and studied the various types of interactions of SAIL molecules with Triton X-100. They concluded the formation of mixed micelles by the incorporation of octylsulphate anion present in SAIL molecules into TX-100 micelle. Wang et al. (2017) investigated the surface-active as well as aggregation behavior of mixed systems constituting SAIL molecules i.e., 1-decyl-3- methylimidazolium bromide, C_10_mimBr, 1-dodecyl-3-methylimidazolium bromide, C_12_mimBr, 1-hexadecyl-3- methylimidazolium bromide, C_16_mimBr, and non-ionic surfactant Triton X-100 in aqueous media. The study revealed the presence of two types of interactions such as hydrogen bonding as well as hydrophobic interactions within the molecules of SAILs and surfactant Triton X-100. Zhang et al. (2010) demonstrate the alterations induced in the micellization behavior of non-ionic surfactant Triton X-100 by adding 1-dodecyl-3-methylimidazolium tetrafluoroborate [C_12_mim][BF_4_] in its aqueous solution. The magnitude of CMC increased dramatically with the addition of [C_12_mim][BF_4_] and the formation of mixed micelles takes place.

There are a lot of other types of ILs such as pyrrolidinium-based, phosphonium-based, or quinolinium-based, which can lead to further new types of ionic liquid-surfactant systems, which can be utilized to investigate the mixed micellization behavior.

## Concluding Remarks

The literature on aggregation behavior of imidazolium-based ILs, SAILs, and surfactants have been reviewed. SAILs can either self-aggregate in the aqueous solution to form micelles or they can form mixed micelles by aggregating in the presence of other surface-active molecules. The mixed micelles have shown greater stability as well as enhanced applicability in comparison to ordinary micelle. Also, the properties of such mixed systems can be modified according to the requirement by just varying the composition of surface-active monomers as well as the temperature of the system. The nature of interactions present in mixed micellar systems can be understood by analyzing mixed micellar parameters obtained by applying various theoretical models. It can be observed from the previous reports that the ILs having longer alkyl chains present in their structure either in the cationic part or in the anionic part show greater surface-activity and lower value of CMC. Also, the temperature has a significant effect on the CMC value as well as on the shape of aggregate formed. The vast variety of ILs and surfactants available provides the opportunity of combining them in various ways. This article could contribute to finding more areas, which require further investigation. Thus, an effort has been made to systematically scrutinize the self-aggregation as well as mixed micellization behavior of a variety of systems. The proper knowledge of the kind of alterations induced in the aggregation behavior upon mixing imidazolium-based ILs with surfactants and the type of interaction prevailing among different constituents of mixed systems can contribute to utilizing such systems in place of conventional surfactants with better reproducibility as well as performance.

## Author Contributions

HK and GK contributed to design of the article. GK organized and wrote the manuscript. All authors contributed to manuscript revision, read, and approved the submitted version.

## Conflict of Interest

The authors declare that the research was conducted in the absence of any commercial or financial relationships that could be construed as a potential conflict of interest.
